# Genetic diversity of avocado from the southern highlands of Tanzania as revealed by microsatellite markers

**DOI:** 10.1186/s41065-020-00150-0

**Published:** 2020-09-14

**Authors:** Ibrahim Juma, Mulatu Geleta, Agnes Nyomora, Ganapathi Varma Saripella, Helena Persson Hovmalm, Anders S. Carlsson, Moneim Fatih, Rodomiro Ortiz

**Affiliations:** 1 Department of Plant Breeding, Swedish University of Agricultural Sciences, Box 101, Växtskyddsvägen 1, 23053 Alnarp, Sweden; 2 Department of Botany, University of Dar es Salaam, Box 35060, Uvumbuzi road, Dar es Salaam, Tanzania

**Keywords:** Breeding, Genetic admixture, Germplasm management, SSR markers, Population structure

## Abstract

**Background:**

Avocado is an important cash crop in Tanzania, however its genetic diversity is not thoroughly investigated. This study was undertaken to explore the genetic diversity of avocado in the southern highlands using microsatellite markers. A total of 226 local avocado trees originating from seeds were sampled in eight districts of the Mbeya, Njombe and Songwe regions. Each district was considered as a population. The diversity at 10 microsatellite loci was investigated.

**Results:**

A total of 167 alleles were detected across the 10 loci with an average of 16.7 ± 1.3 alleles per locus. The average expected and observed heterozygosity were 0.84 ± 0.02 and 0.65 ± 0.04, respectively. All but two loci showed a significant deviation from the Hardy-Weinberg principle. Analysis of molecular variance showed that about 6% of the variation was partitioned among the eight geographic populations. Population F_ST_ pairwise comparisons revealed lack of genetic differentiation for the seven of 28 population pairs tested. The principal components analysis (PCA) and hierarchical cluster analysis showed a mixing of avocado trees from different districts. The model-based STRUCTURE subdivided the trees samples into four major genetic clusters.

**Conclusion:**

High diversity detected in the analysed avocado germplasm implies that this germplasm is a potentially valuable source of variable alleles that might be harnessed for genetic improvement of this crop in Tanzania. The mixing of avocado trees from different districts observed in the PCA and dendrogram points to strong gene flow among the avocado populations, which led to population admixture revealed in the STRUCTURE analysis. However, there is still significant differentiation among the tree populations from different districts that can be utilized in the avocado breeding program.

## Background

Avocado (*Persea americana* Mill.) is a highly heterozygous diploid species with 12 pairs of chromosomes [1]. It produces edible, nutritious and commercially important fruits. *Persea americana* is a polymorphic species with numerous taxa that are adapted to different climates and altitudinal ranges. These taxa are considered to be botanical varieties [2] and include *P. americana* var. drymifolia, *P. americana* var. guatemalensis and *P. americana* var. americana [3], which are commonly referred to as the Mexican, Guatemalan and West Indian horticultural races, respectively [4]. Avocado is a cross-pollinating species with a reported outcrossing ranging from 74 to 96% [5]. The three avocado races are cross-compatible, and hybridisation can occur between trees of different races when grown near each other [6, 7].

Microsatellites are DNA sequences with 2 to 10 base pair repeat motifs, typically repeated 5 to 50 times [8, 9]. They are spread throughout the genome, especially in the euchromatic regions of eukaryotic chromosomes, both in the coding and non-coding DNA regions [10, 11]. Microsatellite regions have a higher mutation rate than other genomic regions leading to a high genetic diversity [12]. Microsatellites are also referred to as simple sequence repeats or SSRs [13]. Polymerase strand-slippage in DNA replication or recombination errors may result in differences in the number of repeats of a given motif (SSR locus) leading to new alleles at the locus under consideration. Thus, different alleles may exist at a given SSR locus, a characteristic that makes the SSRs more informative than other molecular markers, including single nucleotide polymorphisms or SNPs [14]. Being highly nformative, codominant, multi-allelic, highly reproducible and transferable among related species, SSR genetic markers have been widely used for estimating gene flow, diversity, crossing over rates and evolution for uncovering intraspecific genetic relatedness [13–16]. They have also been used in linkage map construction, for quantitative trait loci (QTL) mapping and marker assisted selection, for DNA fingerprinting of cultivars and for estimation of the degree of kinship between genotypes [17, 18].

Genetic diversity refers to differences in genomic regions among individuals, populations and species. It allows populations to adapt to environmental changes. The wider the genetic diversity, the higher the chance of individuals harbouring allele variants that can help cope with given environmental changes. Such individuals will survive and transfer the favourable alleles to their offspring [19].

In Tanzania, avocado is one of the most important commercial fruits sold on domestic and international markets [20]. The first report of avocado cultivation in Tanzania dates back to 1892 [21, 22]. The crop has been grown, mainly seed propagated, for over 100 years and has adapted to a wide range of topography, habitats and climates. As a result, a large diversity has accumulated in this germplasm. So far, only a single study has been conducted to assess the diversity of this germplasm using morphological traits [23]. The aim of the present study was to uncover the genetic diversity of this germplasm using microsatellite markers. The results from the study can be used to establish proper management and conservation strategies and for future breeding of the crop.

## Results

### Microsatellite polymorphism and diversity

A total of 167 different alleles were recorded for the 10 loci across the 226 sampled avocado trees. The mean number of alleles/locus for all loci was 16.70 ± 1.30 (Table 1). All markers were polymorphic and detected at least 10 alleles each. The highest number of alleles per locus was 23 (AVAG22) followed by 20 (LMAV02 and LMAV29). The lowest number of alleles was 10, which was detected for locus LMAV35. The effective number of alleles ranged from 3.84 (AVAG05) to 9.59 (AVAG22) with an average of 6.81 ± 0.66. The Shannon’s information index (I) ranged from 1.69 (LMAV35) to 2.59 (AVAG22). The minimum and maximum observed heterozygosity was 0.46 (LMAV14) and 0.82 (LMAV31), with an average of 0.65 ± 0.04. The average polymorphism information content was 0.82 ± 0.02 and it spanned from 0.70 (AVAG05) to 0.89 (AVAG22). With the exception of LMAV29 and LMAV31, the loci showed a significant deviation from the Hardy-Weinberg equilibrium.

**Table 1:** Diversity in avocado trees grown from seeds in eight districts in southern highlands of Tanzania

Locus name	Repeats	Na	Ne	Ho	He	I	HW	PIC
*AVAG05*^*a*^	(AG)_10_	17	3.84	0.54	0.74	1.73	***	0.70
*AVAG22*^*a*^	(GA)_15_	23	9.59	0.71	0.90	2.59	***	0.89
*AVMIX01*^*a*^	(AT)_7_,(AG)_12_	16	8.96	0.68	0.89	2.40	***	0.88
*ESTAVGA03*^*b*^	(TC)_20_	14	8.32	0.78	0.88	2.31	***	0.87
*LMAV02* ^*b*^	(AC)_8_(AG)_14_	20	6.81	0.54	0.85	2.30	***	0.84
*LMAV14* ^*b*^	(AGAGGG)_4_(AG)_3_	19	7.94	0.46	0.87	2.33	***	0.86
*LMAV24* ^*b*^	(AG)_15_	11	4.34	0.69	0.77	1.79	*	0.75
*LMAV29* ^*b*^	(CTT)_8_(CT)_11_	20	7.35	0.67	0.86	2.32	NS	0.85
*LMAV31* ^*b*^	(GA)_21_	17	7.02	0.82	0.86	2.20	NS	0.84
*LMAV35* ^*b*^	(GAA)_5_(GA)_14_	10	3.99	0.62	0.75	1.69	*	0.72
Mean		16.70	6.81	0.65	0.84	2.17		0.82
Standard error		1.30	0.66	0.04	0.02	0.10		0.02

*, ** and *** indicate significance of *p* value at *p* < 0.05, ≤0.01 and ≤ 0.001, respectively whereas NS indicates the non-significance of the *p* value after Bonferroni correction. ^a^ = from [24] ^b^ = from [25]

Na: Observed number of alleles

Ne: Effective number of alleles [26]

Ho: Observed heterozygosity

He: Nei’s [27] expected heterozygosity

I: Shannon information index [28]

HW: Hardy-Weinberg equilibrium test

PIC: Polymorphism information content

### Genetic diversity among the eight avocado geographic populations

Analysis of genetic diversity of the 226 avocado trees at the intra-population level revealed that for the average observed number of alleles (Na), the Njombe urban population recorded lowest value i.e., 4.20 ± 0.36, whereas both Mbeya rural and Njombe rural populations recorded peak value, i.e., 10.70 ± 2.26 and 10.70 ± 0.70, respectively (Table 2). The mean value of effective number of alleles (Ne) was lowest (2.96 ± 0.29) in the Njombe urban and highest (5.93 ± 0.57) in the Mbozi population. For the Shannon’s information index (I), the Njombe rural population had the highest average value, 1.96 ± 0.09, whereas the Njombe urban had the lowest value, 1.19 ± 0.09. The Wanging’ombe and Njombe urban populations had the lowest average values for the observed (Ho) and the expected (He) heterozygosity, i.e., 0.51 ± 0.06 and 0.71 ± 0.04, respectively. The highest value for Ho was reported in Mbozi, i.e., 0.71 ± 0.10, whereas for He, the highest value was reported in Mbozi and Njombe rural, i.e., 0.83 ± 0.03 and 0.83 ± 0.02, respectively. The lowest and highest average gene diversity was detected in Njombe urban (0.47 ± 0.09) and Mbeya rural (0.65 ± 0.11).

**Table 2:** Diversity information among the eight geographic populations (districts)

District	Na	Ne	He	Ho	I	Gene diversity
Mbeya city	9.60 ± 2.68	4.98 ± 0.34	0.80 ± 0.02	0.70 ± 0.04	1.83 ± 0.08	0.63 ± 0.10
Mbeya rural	10.70 ± 2.26	5.86 ± 0.68	0.82 ± 0.03	0.67 ± 0.04	1.95 ± 0.10	0.65 ± 0.11
Rungwe	7.20 ± 0.63	4.10 ± 0.47	0.73 ± 0.04	0.59 ± 0.08	1.54 ± 0.12	0.54 ± 0.09
Busokelo	5.78 ± 0.61	3.46 ± 0.48	0.73 ± 0.03	0.70 ± 0.05	1.29 ± 0.17	0.59 ± 0.10
Njombe urban	4.20 ± 0.36	2.96 ± 0.29	0.71 ± 0.04	0.60 ± 0.04	1.19 ± 0.09	0.47 ± 0.09
Njombe rural	10.70 ± 0.70	5.90 ± 0.50	0.83 ± 0.02	0.61 ± 0.04	1.96 ± 0.09	0.60 ± 0.32
Wanging’ombe	9.30 ± 0.97	5.67 ± 0.76	0.81 ± 0.03	0.51 ± 0.06	1.86 ± 0.13	0.57 ± 0.10
Mbozi	9.30 ± 0.75	5.93 ± 0.57	0.83 ± 0.03	0.71 ± 0.10	1.92 ± 0.11	0.56 ± 0.09

### Molecular variance and population divergence

Analysis of molecular variance was employed to detect genetic divergence within and among the eight avocado populations. The analysis partitioned 6.08% of the variation among the populations, 17.04% among individuals within populations, and 76.87% within all individuals (Table 3). The detected variations were significant at *P* < 0.0001. The total population differentiation due to genetic structure, i.e., the fixation index (F_ST_), was 0.061 (*P* < 0.0001; Table 3). When the populations were further grouped into regions, 1.98 and 4.71% variation was noticed among groups (regions) and among populations (districts) within groups, respectively. However, the variation among individuals within populations (districts) and within individuals was similar to the values in the analysis performed on the eight populations. AMOVA conducted by grouping the genotypes into four groups according to their altitude of growth revealed that 2.52% of the total variation differentiated the groups (*P* < 0.0001).

**Table 3:** Analysis of molecular variance using 1000 permutations for 226 avocado trees from eight populations (districts)

Source of variation	Sum of squares	Variance component	Percentage variation	F-Statistics	*P*-value
*A: When the sampled trees were grouped according to geographic populations (districts)*					
Among populations	115.33	0.258 Va	6.08	F_ST_ = 0.061	< 0.0001 (Va and F_ST)_
Among individuals within populations	841.00	0.722Vb	17.04	F_IS_ = 0.181	< 0.0001 (Vb and F_IS)_
Within individuals	620.00	3.258Vc	76.87	F_IT_ = 0.231	< 0.0001 (Vc and F_IT)_
Total	1576.33	4.239			
*B: When the geographic populations (districts) were further grouped according to regions (Mbeya, Njombe and Songwe)*					
Among groups (regions)	43.605	0.085 Va	1.98	F_CT_ = 0.019	0.02 (Va and F_CT)_
Among populations (districts) within groups	71.721	0.201 Vb	4.71	F_SC_ = 0.048	< 0.0001 (Vb and F_SC)_
Among individuals within populations (districts)	841.004	0.722 Vc	16.93	F_IS_ = 0.181	< 0.0001 (Vc and F_IS)_
Within individuals	620.000	3.258 Vd	76.38	F_IT_ = 0.236	< 0.0001 (Vd and F_IT)_
Total	1576.330	4.266			
*C: When the sampled trees were grouped in four altitudinal groups*^*1*^ *(719–1200; 1201–1600; 1601–1800; 1801–2136 masl)*					
Among altitudinal groups	45.185	0.107Va	2.52	F_ST_ = 0.025	< 0.0001 (Va and F_ST)_
Among individuals within altitudinal groups	911.144	0.865Vb	20.45	F_IS_ = 0.210	< 0.0001 (Vb and F_IS)_
Within individuals	620.000	3.258Vc	77.03	F_IT_ = 0.230	< 0.0001 (Vc and F_IT)_
Total	1576.330	4.230			

^1^ altitudinal groups were regarded as populations and that is why F_ST_ was calculated

The genetic divergence between the eight avocado populations was established by calculating pairwise F_ST_ comparisons (Table 4). 21 of the 28 pairs of populations showed a significant differentiation (*P* ≤ 0.05), with their F_ST_ values ranging from 0.0111 (Mbeya city and Mbeya rural) to 0.1475 (Rungwe and Mbozi). The second highest F_ST_ value (0.1369, *P* < 0.05) was recorded for Njombe urban and Mbozi.

**Table 4:** Population pairwise F_ST_ comparisons between eight avocado populations

Population	Mbeya city	Mbeya rural	Rungwe	Busokelo	Njombe urban	Njombe rural	Wanging’ombe	Mbozi
Mbeya city	0.0000							
Mbeya rural	0.0111*	0.0000						
Rungwe	0.0653*	0.0329*	0.0000					
Busokelo	0.0522*	0.0254*	0.0076	0.0000				
Njombe urban	0.0768*	0.0446*	−0.0057	−0.0349	0.0000			
Njombe rural	0.0323*	0.0215*	0.0354*	0.0231*	0.0265	0.0000		
Wanging’ombe	0.0321*	0.0249*	0.0341*	0.0062	0.0057	0.0060	0.0000	
Mbozi	0.0521*	0.0583*	0.1475*	0.1290*	0.1369*	0.0477*	0.0670*	0.0000

* indicates significance level at *P* ≤ 0.05 for the tested values

### Principal components analysis and hierarchical cluster analysis

**Fig. 1:**
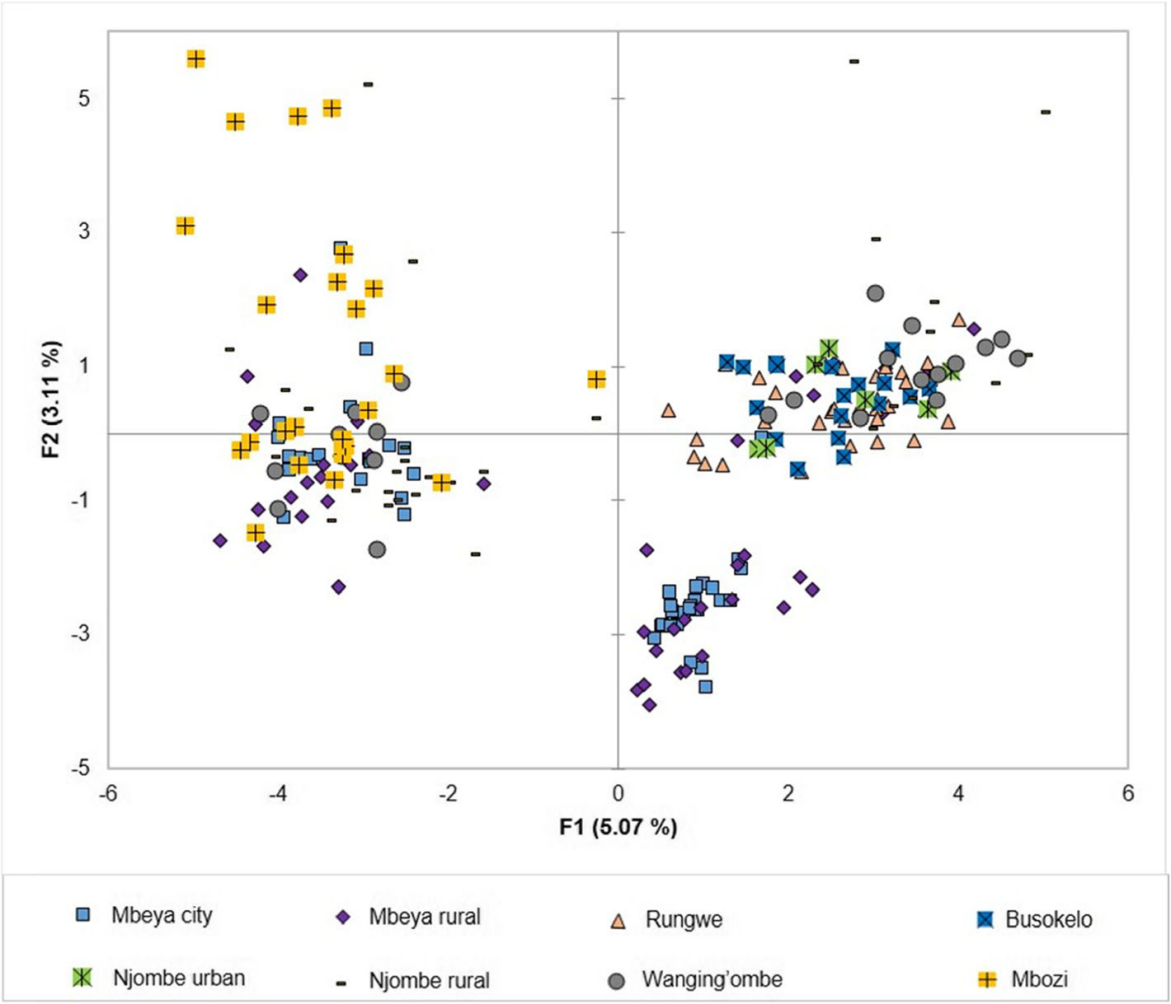
PCA showing the genetic relationships among the 226 avocado trees

Principal components analysis (PCA) was used to study the genetic relationships among the 226 avocado trees (Fig. 1). While the first two axes of the PCA accounted for 8.18% of all variation, most of the trees grouped irrespective of geographic origin. The sampled trees from Njombe urban tend to group to the right and so do those from Busokelo and Rungwe. The trees from the remaining populations are quite scattered in the plot. Grouping of samples from different districts or regions in the PCA plot points to genetic admixture among the sampled trees.

**Fig. 2:**
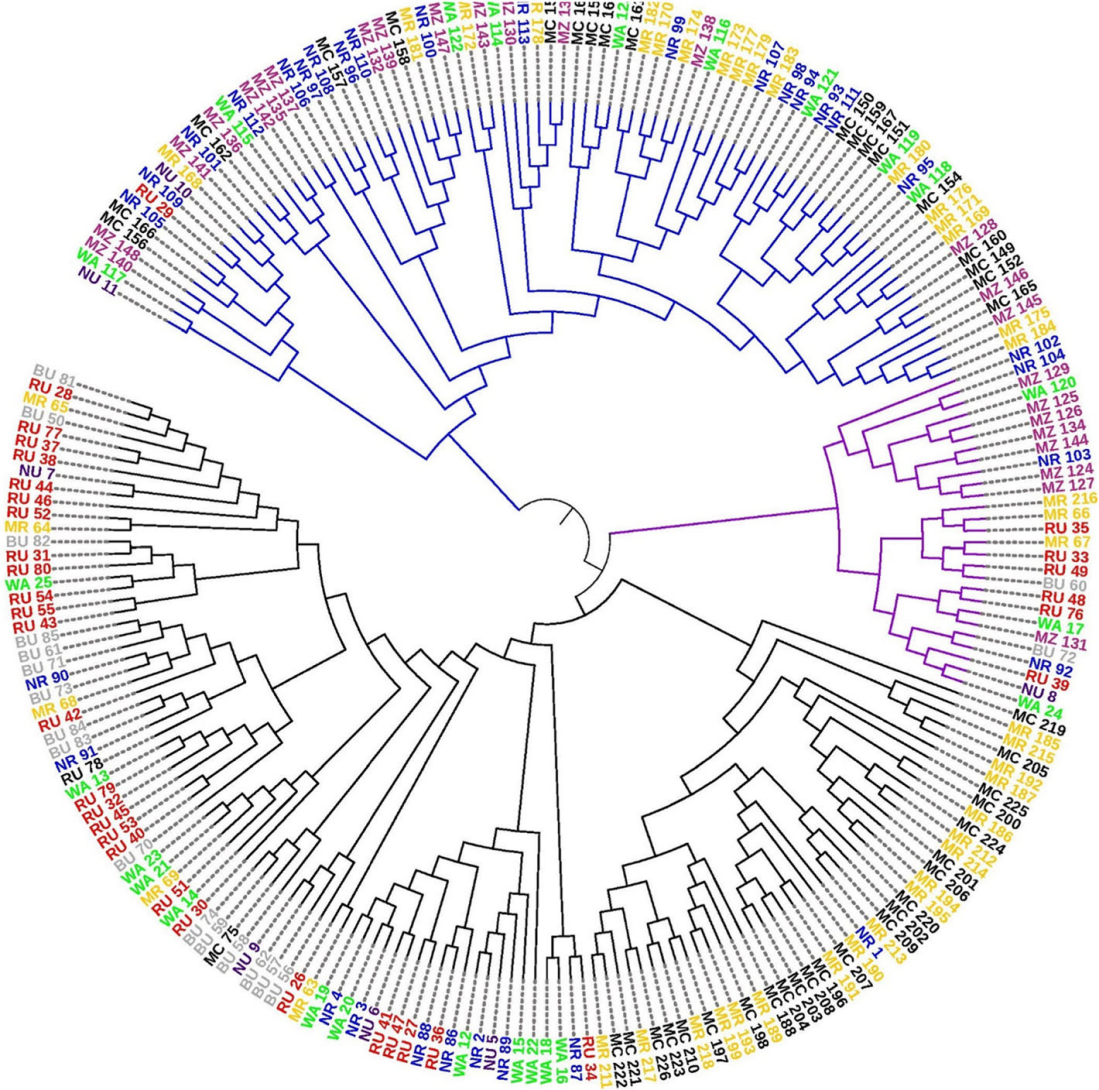
Dendrogram of the 226 avocado trees constructed with UPGMA showing genetic relationships between the analysed samples. Samples collected from a common district are represented by names in the same text colour; black = Mbeya city, yellow = Mbeya rural, red = Rungwe, grey = Busokelo, dark purple = Njombe urban, blue = Njombe rural, green = Wanging’ombe and shining purple = Mbozi

The genetic distance matrix of the 226 avocado tree samples was used to study the genetic relationships among the eight populations through hierarchical clustering. The UPGMA-based dendrogram produced three major groups, each containing samples from different districts and regions (Fig. 2), pointing at genetic admixture between samples from different districts.

### Population structure and genetic relationship of the studied avocado samples

**Fig. 3:**
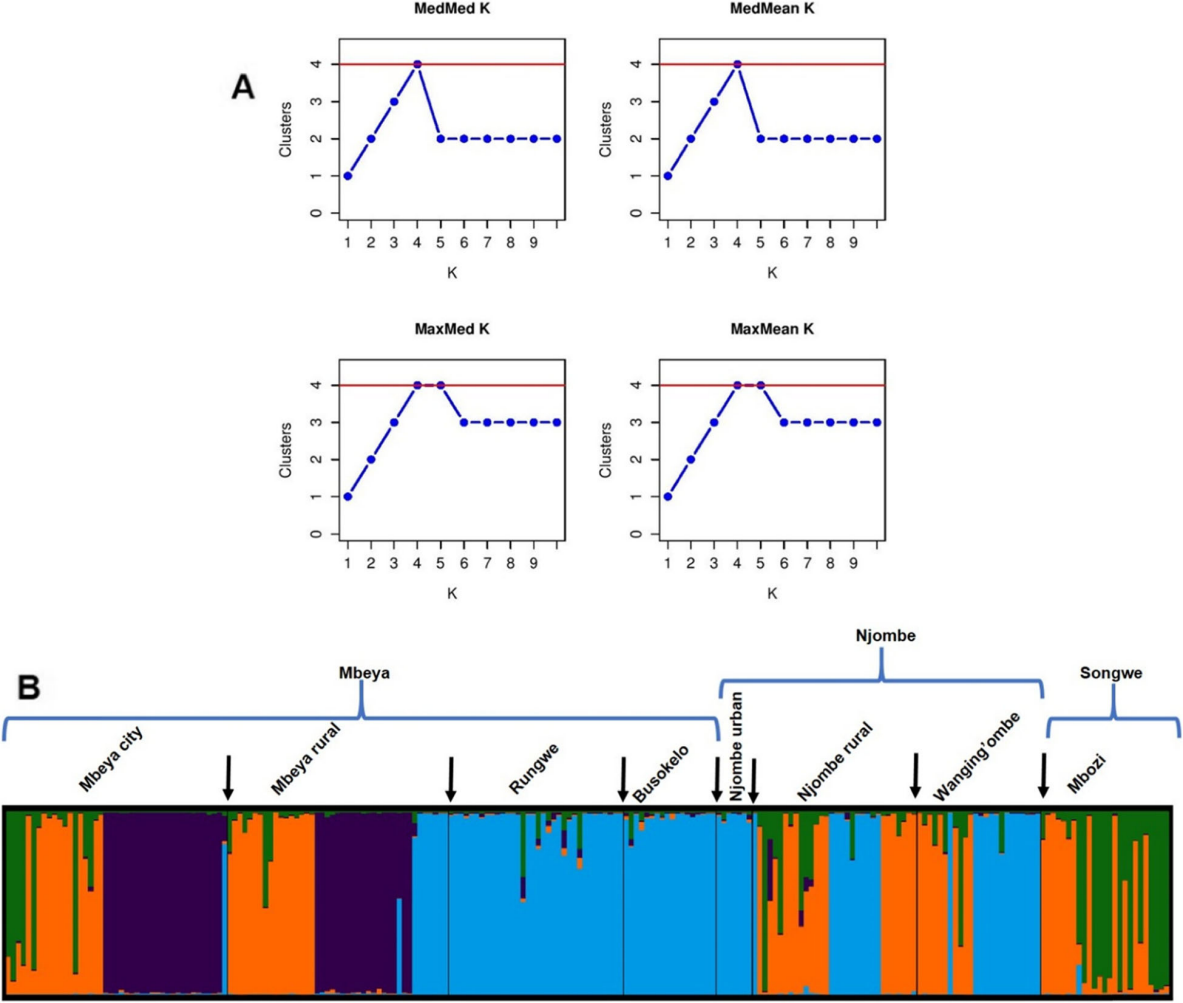
Population structure of the analysed trees. **a:** Identification of the optimum K-value using four different approaches developed by Puechmaille [29]; **b:** Population structure of 226 avocado trees suggesting varying levels of genetic admixture from four origins (colours) in the eight districts in the southern highlands of Tanzania

Estimation of K-values, based on the methods by Puechmaille [29], revealed that the most probable K-value for our genetic data set was four (MedMeaK, MaxMeaK, MedMedK, MaxMedK = 4; Fig. 3a). This proposes that the 226 avocado tree samples can be clustered into 4 subpopulations or clusters (Fig. 3b). The genetic structure suggests a high similarity between the Busokelo and Njombe urban avocado populations as well as between the Wangingo’mbe and Njombe rural populations. Members of Mbozi population showed highly similar genetic constitution with some members of populations from other districts, such as Mbeya city and Mbeya rural across the 10 SSR loci.

## Discussion

In the present study, a total of 167 alleles were detected using 10 SSR loci across 226 sampled avocado trees with the number of alleles ranging from 10 to 23 per locus (Table 1). For comparison, Schnell et al. [30] detected 256 alleles using 14 SSR loci across 428 plants, with number of alleles ranging from 8 to 30 per locus. The average number of alleles per locus recorded in the present study was 16.7. A higher number of alleles per locus has been reported by Schnell et al. [30] and Guzmán et al. [31], 18.8 and 19.5, respectively. Gross-German and Viruel [25] and Abraham and Takrama [32] reported lower numbers, 11.4 and 11.5, respectively. Similarly, Liu et al. [33] reported the lowest number of alleles per locus, 3.10, across 10 SSR loci for 56 avocado trees investigated in Hainan Province, China. The differences between our results and the previously reported results could be due to variation in levels of polymorphism of the markers used, sample size, the diversity of the germplasm investigated and the platforms employed for resolution of amplified products [34]. The quality of genomic DNA used in PCR amplification, optimization of PCR protocols and differences in allele scoring accuracy could also be accounted for these differences. The 16.7 alleles per locus detected in the present study is comparable to that reported from other cross-pollinated species like maize (21.7 alleles [35]) and bur oak (14.3 alleles [36]).

The average observed heterozygosity for the 10 SSR loci obtained in the present study was 0.65 (Table 1) which is similar to 0.64 and 0.61 obtained by Schnell et al. [30] and Guzmán et al. [31], respectively, indicating similar levels of genetic diversity for their analysed samples. Lower observed heterozygosity have been reported by Abraham and Takrama [32]: 0.48, Boza et al. [37]: 0.56, and Liu et al. [33]: 0.39, thereby pointing to a lower genetic diversity in the germplasm used or differences in the polymorphism levels of the SSR loci used by them vis-à-vis ours.

Expected and observed heterozygosity, Shannon’s information index and average gene diversity are indicators of the extent of genetic diversity in populations. The analysis of diversity at the intra-population level revealed that while the observed heterozygosity, the Shannon’s information index and average gene diversity were highest for the Mbozi, Njombe rural and Mbeya rural populations, respectively, the excepted heterozygosity was highest for both Mbozi and Njombe rural (Table 2). This suggests that the Mbozi, Njombe rural and Mbeya rural populations are more diversified than the other populations and thus may offer elite materials for breeding programmes [38]. The three populations may also be able to cope with changes in environmental conditions in a better way than the other populations [19]. All the four diversity measures, except observed heterozygosity, were lowest in the Njombe urban population pointing to lower genetic diversity. This result might be attributed to the massive replacement of local seed propagated avocado with the commercial cultivars leading to a decreasing variation within the genepool. The lowest observed heterozygosity detected in Wanging’ombe, relative to the Njombe urban population, could possibly be attributed by the presence of null alleles and linkage disequilibrium. However, it is worth noting that the findings of the four diversity measures at intra-population could be affected by variation in sample size among the eight populations.

Most of the loci in the present study showed significance deviation from the Hardy-Weinberg equilibrium (Table 1). Geographical structure and inbreeding within subpopulations may lead to Hardy-Weinberg equilibrium deviations. Wright’s fixation indices (F_IT_, F_ST_, and F_IS_) can be used to assess these within- and among-population components of genetic variation. F_IT_ measures the excess (F_IT_ > 0; heterozygosity deficit) or deficit (F_IT_ < 0: heterozygosity excess) of homozygotes at the global level [39]. The global heterozygosity deficit (F_IT_), when AMOVA was calculated without considering regions, was 0.231 (*P* < 0.0001; Table 3). This implies that the observed homozygotes exceeded the expected value by about 23%. In other words, there was a reduction by 23% of observed heterozygotes relative to the expected ones. The result may suggest that avocado has a significant level of self-pollination although it is generally regarded as an out-crossing species. The SSR loci showing significant deviation from HWE could be in linkage disequilibrium with genic loci under selection in the form of heterozygote disadvantages. The fixation index, F_IS_, is an inbreeding coefficient that measures the excess (F_IS_ > 0; heterozygosity deficit) or deficit (F_IS_ < 0: heterozygosity excess) of homozygotes within a subpopulation [39]. The average inbreeding coefficient of individuals within subpopulations (districts) (F_IS_) was 0.181 (*P* < 0.0001), which indicates that within subpopulations, the observed homozygotes exceeded the expected value by about 18%. In other words, there was a reduction by 18% of observed heterozygotes relative to the expected ones within subpopulations. At the global level, this implies that about 78% (i.e., FIS*100%/FIT) of the global heterozygosity deficit was due to the within population deficit. F_ST_ is the fixation index that measures differentiation between subpopulations and range from 0 to 1. A value of 0 indicates that the populations under consideration are interbreeding freely (complete panmixis), while a value close to 0 indicates an unstructured population [40]. A value of 1 infers that all genetic variation is explained by the population structure, and that the populations under consideration do not share any genetic diversity [40]. In the present study, the global degree of genetic differentiation (F_ST_) for the eight avocado geographical populations was 0.061 (*P* < 0.0001; Table 3). This indicates that there is a significant district-based subdivision of Tanzanian avocados. This is possibly the results of mutations or genetic drift and indirect selection pressure that normally lead to lose of certain alleles or change in allele frequencies. As shown through analysis of molecular variance (AMOVA; Table 3) about 94% of the genetic variation was shared by the eight populations. The F_ST_ value detected in our study was lower than the 0.19, 0.22 and 0.25 previously reported by Boza et al. [37], Guzmán et al. [31] and Gross-German and Viruel [25], respectively. This could be due to the fact that our study was based on samples collected from only local avocados (excluding commercial cultivars) and sampling involved only three nearby geographic regions in Tanzania. Contrary to our study, Boza et al. [37] studied avocado samples from United States and Mexico representing *Persea americana* (218 samples), *P. nubigena* (2 samples) and *P. kruguii* (1 sample). On the other hand, Guzmán et al. [31] analysed only *P. americana* collected in one country comprising local selections, root stocks and commercial cultivars. Similarly, the 315 samples analysed by Gross-German and Viruel [25] included also 5 samples from *P. longipes, P. nubigens* and *P. schiedeana*. However, the average F_ST_ in the present study is comparable to the 0.05 reported by Cañas-Gutiérrez et al. [15] for 197 avocado samples from Colombia.

Analysis of molecular variance showed that 6.08% of the total genetic variation was partitioned among the 8 districts (Table 3). When the samples were grouped according to elevation ranges, the genetic variation among groups was 2.52%. These results concur with that of Teshome et al. [41], who observed lower differentiation among altitude-based groups of Ethiopian field pea (*Pisum sativam* L.) populations (2%) compared to that of region-based groups (8%).

In the present study, principal components analysis (PCA) and hierarchical cluster analysis were used to study the genetic relationships among the sampled trees. Neither the PCA nor the dendrogram separated these trees according to their districts or regions. This was in line with AMOVA findings (Table 3) which showed that about 94% of the total genetic variation was shared by the populations. The PCA and dendrogram findings are also supported by the population pairwise F_ST_ that revealed absence of differentiation between pairs of populations, such as Rungwe versus Njombe urban and Buokelo versus Njombe urban. Similar results were obtained when these avocado trees were characterised with morphological markers [23]. Genetic admixture among the avocado populations is attributed to sharing of seeds between farmers from different districts and selling of avocado produce from one district to another where the seeds could then be planted. It may also be due to introduction of highly similar germplasm to more than one districts/regions.

The model-based STRUCTURE was used to study the population structure of the 226 avocado plants. The results showed that the sampled plants can be regrouped into four clusters based on their genetic characteristics detected at the 10 studied loci. High similarity in population structure was noticed between the Busokelo and Njombe urban avocados as well as the Wangingo’mbe and Njombe rural avocados. This result is supported by lack of differentiation among these pairs as shown by the population pairwise F_ST_ values (Table 4). Each geographic population (district) have alleles originated in at least three STRUCTURE based populations (clusters). Various analyses such as AMOVA, global F_ST_, PCA and UPGMA revealed that avocados grown in different districts of Tanzania show high genetic similarity with low but significant genetic differentiation between them.

## Conclusion

High diversity was detected in the analysed avocado germplasm based on standard and molecular diversity indices. These findings implies that this germplasm is a potentially valuable source of variable alleles that might be harnessed for genetic improvement of this crop in Tanzania. The principal components analysis and hierarchical cluster analysis showed a mixing of avocado trees from different districts, pointing to strong gene flow among the eight populations. This is in line with the results of the model-based population structure analysis that revealed that the alleles of each district based populations originated from at least three of the four genetic populations.

## Materials and methods

### Collecting samples and DNA extraction

**Fig. 4:**
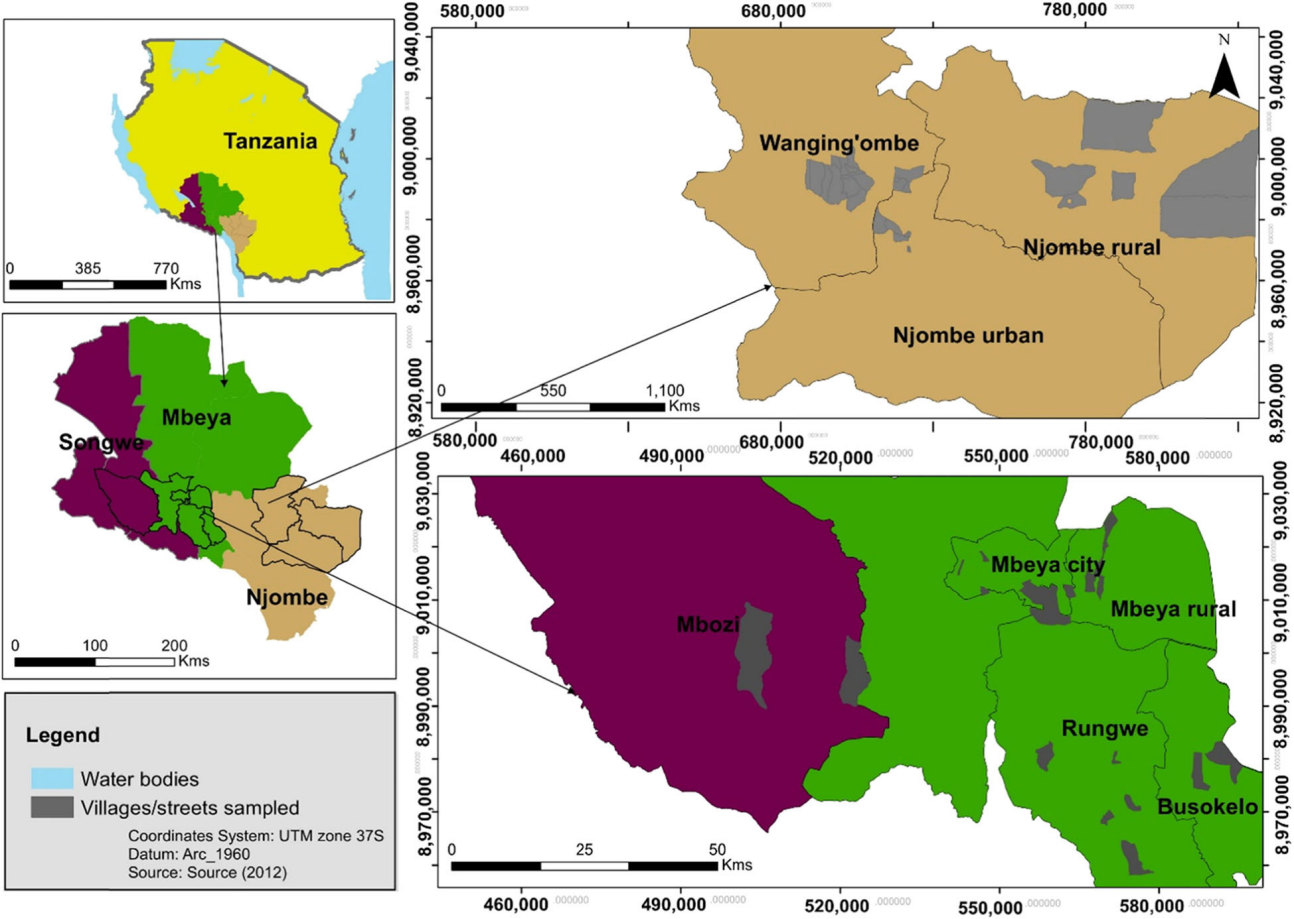
Location of sampling sites. (1) Top left: Tanzania map displaying location of the three avocado-rich regions; (2) Bottom left: regions showing locations of the eight sampled districts; (3) Top and bottom right: districts showing locations of villages/streets where sampling was carried out

Samples were collected in three regions; i.e., Mbeya, Njombe and Songwe, which are located in the southern highlands of Tanzania. From these regions a total of 41 villages or streets were visited across eight districts that are renowned for harbouring many seed propagated avocado trees. Locations of the study sites are given in Fig. 4. The number of trees sampled in each district and region is presented in Table 5 whereas some data on climate of the studied districts are presented on Table 6.

**Table 5:** Number of trees sampled in each district

Region	District	Number of trees sampled
Mbeya	Mbeya city	43
	Mbeya rural	43
	Rungwe	34
	Busokelo	18
Njombe	Njombe urban	7
	Njombe rural	32
	Wanging’ombe	24
Songwe	Mbozi	25
Total number trees		226

**Table 6:** Climate data of the eight districts

Region	District	Climate	Climate classification according to Köppen and Geiger	Average temperature for a year	Minimum monthly average temperature	Maximum monthly average temperature	Source
Mbeya	Mbeya city	Mild, and generally warm and temperate	Cwb	17.7 °C	14.6 °C (July)	20.1 °C (November)	Climate-data.org (Undated-**a**) [42]
	Mbeya rural	NIL	NIL	NIL	NIL	NIL	
	Rungewe (Tukuyu)	Oceanic subtropical highland climate	Cwb	20.5 °C	17.2 °C (July)	23.1 °C (November)	Weatherbase (Undated-**a**) [43]
	Busokelo (Masoko)	Tropical monsoon climate	Am	22.5 °C	19.7 °C (July)	24.9 °C (November)	Climate-data.org (Undated-**b**) [44]
Njombe	Njombe urban	Oceanic Subtropical Highland Climate	Cwb	18.1 ° C	14.9 ° C (July)	20.6 °C (November)	Weatherbase (Undated-**b**) [45]
	Njombe rural	NIL	NIL	NIL	NIL	NIL	
	Wanging’ombe	Mild, and generally warm and temperate	Cwa	20.2 °C	17.7 °C (July)	22.5 °C (December)	Climate-data.org (Undated-**c**) [46]
Songwe	Mbozi (Mlowo)	Warm and temperate	Cwb	19.5 °C	17.1 °C (July)	21.5 °C (October)	Climate-data.org (Undated-**d**) [47]

We visited the study sites from February to August in 2017 and young, leaf samples were collected from a total of 226 seed-propagated avocado trees. We sampled four to six leaves from each tree and then packed them in porous tea bags (two to three leaves per bag). The bags were then put into plastic bags followed by addition of silica gel to dry the leaves. When needed, we replaced the worn-out silica gel and continued doing so until the leaf samples were completely dry. Complete dryness was determined as when added silica gel retained its original colour. We extracted DNA from the dried young avocado leaf samples using the Thermo Scientific Genomic DNA Purification Kit following the manufacturer’s instructions with minor modifications. DNA integrity was checked in a 1.2% agarose gel electrophoresis and its quality and quantity was assessed with a NanoDrop Spectrophotometer.

### Microsatellite analysis

Forty microsatellite markers were selected among those developed by Sharon et al. [24] and Gross-German and Viruel [25] based on their reported levels of polymorphism. The primers were tested on eight avocado samples from eight populations, each representing a different district. Thereafter, 16 microsatellite markers that were highly polymorphic within the eight samples were chosen and used for analysis of all samples. However, only 10 of the 16 markers showed consistent amplification and the data analysis is therefore based on these 10 markers. Background information about the microsatellites, such as names and repeat motifs, is presented in Table 2. Amplification of target microsatellite loci was carried out in a total reaction volume of 25 μl containing 2.5 μl of 10X PCR buffer, 1.5 μl of 25 mM MgCl_2_, 0.3 μl of 25 mM dNTPs, 0.75 μl of 10 μM of each forward and reverse primer, 0.2 μl of 5 U/μl Taq polymerase and 25 ng genomic DNA. PCR reactions were run in a S1000™ thermal cycler (BIO RAD, Hercules, CA, USA) using a program of initial denaturation at 94 °C for 1 min, 35 cycles of 1 min denaturation at 94 °C, 30 s annealing depending on the specific primers’ annealing temperature and 1 min extension at 72 °C, followed by a 10 min final extension at 72 °C. The size of the PCR products were determined using an Applied Biosystems 3500 Genetic Analyzer (Thermo Fisher Scientific, Waltham, MA, USA).

### Data analysis

Standard diversity indices for markers; i.e., observed and effective number of alleles, observed Nei’s [27] and expected heterozygosity and Shannon’s information index [28] were computed using Popgene32 software version 1.32 [48]. Polymorphism information content for each SSR locus was assessed using Cervus version 3.0.7 [49]. Hardy-Weinberg equilibrium tests (molecular diversity index) were carried out in Popgene32 software version 1.32 [48]. To assess diversity at intra–population level, we computed standard diversity indices for each geographical population (district) using Arlequin 3.5.2.2 [50]. The indices calculated were observed number of allele and observed and expected heterozygosity. The effective number of alleles and Shannon’s information index were computed using GenAlEx version 6.5 [51]. We also estimated average gene diversity across all loci for each population in Arlequin 3.5.2.2.

Analysis of molecular variance (AMOVA) was carried out in Arlequin 3.5.2.2 under 1000 permutations, 100,000 steps in Markov chain and 10,000 Dememorisation Steps. Hierarchical global AMOVA was conducted by grouping individual samples according to districts, regions and elevation ranges. Arlequin 3.5.2.2 was used to compute fixation indices (F_ST_, F_IT_, F_IS,_ F_CT and_ F_SC_) and pairwise comparisons between the eight geographical populations.

We employed principal components analysis (PCA) to display the genetic relationships among the eight avocado geographical populations (districts). Allele composition for each tree was computed in the adegenet R package [52] and then used in PCA in XLSTAT version 2019.4.2 [53]. Hierarchical cluster analysis was also deployed to assemble avocado samples with similar genetic characteristics across the 10 loci in the same group. In order to achieve this, we computed Nei’s genetic distance in GenAlEx and imported it in MEGAX [54] where the dendrogram in the newick format was produced using the unweighted pair group method with arithmetic mean (UPGMA) [55]. The dendrogram was visualized and customized in the Interactive tree of life (iTOL) v4 [56]. To identify genetic populations for the 226 avocado trees, we performed a Bayesian cluster analysis on the allele dataset using STRUCTURE 2.3.4 [57–59]. The admixture model was adopted with 10,000 burn-in period and 100,000 Monte Carlo Markov chain iterations. The range of K, the probable number of subpopulations or clusters, tested was 1 to 10. For each K value, we performed 20 independent runs. The structure results were analysed and visualised in STRUCTURE SELECTOR [60] where the optimal number of genetic clusters was computed based on distinct approaches; the median of medians (MedMedK), median means (MedMeaK), maximum of medians (MaxMedK) and maximum of means (MaxMeaK) [29].

## Data Availability

The datasets used and/or analysed during the current study are available from the corresponding author on reasonable request.

## Abbreviations

AMOVA: Analysis of molecular variance
He: Nei’s (1973) expected heterozygosity
Ho: Observed heterozygosity
HW: Hardy-Weinberg equilibrium test
I: Shannon information index (Lewontin, 1972)
Na: Observed number of alleles
Ne: Effective number of alleles (Kimura and Crow, 1964)
PCA: Principal components analysis
PIC: Polymorphism information content
UPGMA: Unweighted pair group method with arithmetic mean
